# L1 Cell Adhesion Molecule (L1CAM) expression in endometrioid endometrial carcinomas: A possible pre-operative surrogate of lymph vascular space invasion

**DOI:** 10.1371/journal.pone.0209294

**Published:** 2018-12-17

**Authors:** Daniela de Freitas, Fernando Nalesso Aguiar, Cristina Anton, Carlos Eduardo Bacchi, Jesus Paula Carvalho, Filomena Marino Carvalho

**Affiliations:** 1 Instituto do Cancer do Estado de São Paulo, Faculdade de Medicina da Universidade de São Paulo, São Paulo, SP, Brazil; 2 Department of Pathology, Faculdade de Medicina da Universidade de São Paulo, São Paulo, SP, Brazil; 3 Consultoria em Patologia, Botucatu, SP, Brazil; 4 Department of Obstetrics and Gynecology, Faculdade de Medicina da Universidade de São Paulo, São Paulo, SP, Brazil; National Cancer Center, JAPAN

## Abstract

**Background:**

Risk stratification of endometrial carcinomas is primarily based on surgical staging that requires extensive retroperitoneal lymph node dissection. One of the most powerful predictor of lymph node involvement is the lymph vascular space invasion (LVSI). The objective of this study was to determine the potential of L1 Cell Adhesion Molecule (L1CAM) to predict LVSI and its association with other risk factors in endometrioid endometrial carcinomas.

**Materials and methods:**

We studied 47 consecutive patients aged 37–88 (61.34±10.52). Twenty-three patients (48.9%) were submitted to complete surgical staging. Nine patients (19.1%) underwent surgical staging without para-aortic dissection. Seven (14.9%) were submitted to hysterectomy with no lymph node dissection. Eight patients (17.0%) only had the biopsy material for analysis. The 32 patients submitted to lymphadenectomy were staged according to the FIGO system and classified among the risk categories of the ESMO-ESGO-ESTRO guidelines. The following histological characteristics were analyzed: tumor size (mm), depth of myometrial infiltration, presence of microcystic, elongated, and fragmented (MELF) pattern of myoinvasion, and lymph vascular space invasion (LVSI). Immunohistochemical analyses of mismatch repair (MMR) proteins MLH1, MSH2, MSH6, and PMS2, p53, and L1CAM were performed in formalin-fixed paraffin embedded whole tumor tissue sections.

**Results:**

LVSI was identified in 26/41 (63,4%) of the cases. L1CAM was positive in 8/47 (17%) cases, all of them positive for LVSI and within the high-risk category of ESMO-ESGO-ESTRO. L1CAM-positive cases were associated with high histological grade and p53 aberrant immunohistochemical profile. Besides, it showed a trend to larger tumors, greater depth of myometrial infiltration, and with a higher frequency of the MELF pattern of myoinvasion. LVSI was also associated with FIGO stage, tumor size, depth of myometrial infiltration, and tumor grade.

**Conclusions:**

L1CAM is highly associated with LVSI and could be used as a pre-operative predictor of lymph node involvement in endometrioid endometrial carcinomas.

## Introduction

The standard treatment for endometrial carcinomas consists of a hysterectomy with bilateral salpingo-oophorectomy and pelvic and para-aortic lymphadenectomy, followed by adjuvant therapy for high-risk disease. Over the past decades debate has developed questioning the need for lymphadenectomy in every case, as well as the criteria for adjuvant treatment. The great challenge is the identification of prognostic biomarkers, preferably to be investigated in biopsy specimens, prior to surgical treatment, to drive toward better therapeutic decisions, including or not the need for lymphadenectomy, and evaluating the benefit of using adjuvant radio- and/or chemotherapy.

Lymph vascular space invasion (LVSI) is one of the most important predictors of lymph node involvement [1–4]. The challenge is how to identify LVSI prior to the primary surgical treatment. A promising biomarker to risk stratification is the L1 Cell Adhesion Molecule (L1CAM), a 200-220kDA transmembrane glycoprotein of the immunoglobulin superfamily that plays an important role in nervous system development, including neuronal migration and differentiation. L1CAM has been shown to be an important prognostic factor in endometrial carcinomas and is predictive of lymph node involvement [5–8].

Because the expression of L1CAM is detectable at the time of the diagnostic biopsy, the main objective of this study is to determine its potential to predict LVSI. If so, L1CAM could be a pre-operatory surrogate of lymph vascular space invasion. In this study, beyond the expression of L1CAM, we also analyze its association with classical risk factors in endometrial carcinomas of endometrioid type, according to the International Federation of Gynecology and Obstetrics (FIGO) stages I-III.

## Materials and methods

### Institutional approval

The study was approved by the Scientific Committee of the Department of Pathology of Faculdade de Medicina da Universidade de Sao Paulo and by the Ethics Committee for Research Projects of the Hospital das Clınicas da Faculdade de Medicina da Universidade de Sao Paulo (Comissao de Ética para Análise de Pesquisa—CAPPesq) (case # 351/16), and Plataforma Brasil (CAAE 59579616.8.0000.0065). It complies with the ethical precepts proposed by the legislation in force in Brazil R466 / 2012. The specific informed consent form for this work was waived by the Ethics Committee above. The decision was based on the fact that the study was retrospective, with use of data from medical records and minimum tissue from paraffin blocks from patients already treated, with no risk or benefit arising from the results, and with guarantees of their anonymity.

### Study cohort

Forty-seven consecutive cases of endometrial carcinomas of endometrioid type from September 2015 to May 2018 were retrieved from the Department of Pathology report files. Patient’s age ranged from 37 to 88 years (61.34±10.52) with a median of 60 years. Twenty-three patients (48.9%) were submitted to complete surgical staging (hysterectomy, bilateral salpingo-oophorectomy, and pelvic and para-aortic lymphadenectomy). Nine patients (19.1%) underwent surgical staging without para-aortic dissection; one of these was submitted to sentinel lymph node biopsy. Seven (14.9%) were submitted to hysterectomy with no lymph node dissection. Eight patients (17.0%) only had the biopsy material for analysis. The 32 patients submitted to lymphadenectomy had 2–109 studied lymph nodes (33.8±25.8; median 32.5). It should be noted that one patient had two sentinel lymph nodes studied. These 32 patients were staged according to the FIGO system [9]. There were 9 (28.1%) FIGO stage IA, 12 (37.5%) FIGO IB, 3 (9.4%) FIGO II, and 8 (25%) FIGO III. Risk classification was performed according to the ESMO-ESGO-ESTRO guidelines [10].

All specimens were analyzed separately by two pathologists (FMC and FNA). The tumors were classified according the WHO 2014 criteria and graded using the FIGO system [11]. The following histological characteristics were analyzed: tumor size (mm), percentage of myometrial infiltration, presence of microcystic, elongated, and fragmented (MELF) pattern of myoinvasion, and lymph-vascular space invasion (LVSI). Immunohistochemistry reactions were performed in 4-μm thick 10% formalin-fixed paraffin-embedded whole tissue sections. Details of the reactions are summarized in Table 1.

**Table 1:** Reagents and methods used for immunohistochemical reactions.

Primary antibody	Clone	Provider	Titration	Antigen retrieval	Time of antigen retrieval
p53	DO-7	Dako*	1:3000	Tris-EDTA pH 9.0	20 min
MLH1	ES05	Dako*	Pure; Linker mouse	Tris-EDTA pH 9.0	20 min
MSH2	G219-1129	Dako*	1:400	Tris-EDTA pH 9.0	30 min
MSH6	SP93	Dako*	1:400; Linker mouse	Tris-EDTA pH 9.0	20 min
PMS2	EP51	Dako*	1:2; Linker mouse	Tris-EDTA pH 9.0	40 min
L1CAM	74-5H7	COVANCE**	1:300	Citrate (pH 6.1)	20 min

*: Dako (Carpinteria, CA, USA)

**: (Covance, San Diego, USA)

Deficiency of DNA mismatch repair (MMR) proteins MLH1, MSH2, MSH6, and PMS2 was defined as complete loss of nuclear expression within tumor cells in the presence of positive internal controls in lymphocytes and/or stroma.

Aberrant p53 protein (mutated/inactivated) was considered if the tumor exhibited strong uniform nuclear staining in >80% of the tumor cells, or the complete absence of such staining in the tumor cells, in the presence of focal nuclear staining of the stromal cells. Wild-type p53 immunostaining was considered if weak or moderate nuclear staining of the tumor cells was present in <80% of tumor cells.

**Fig 1:**
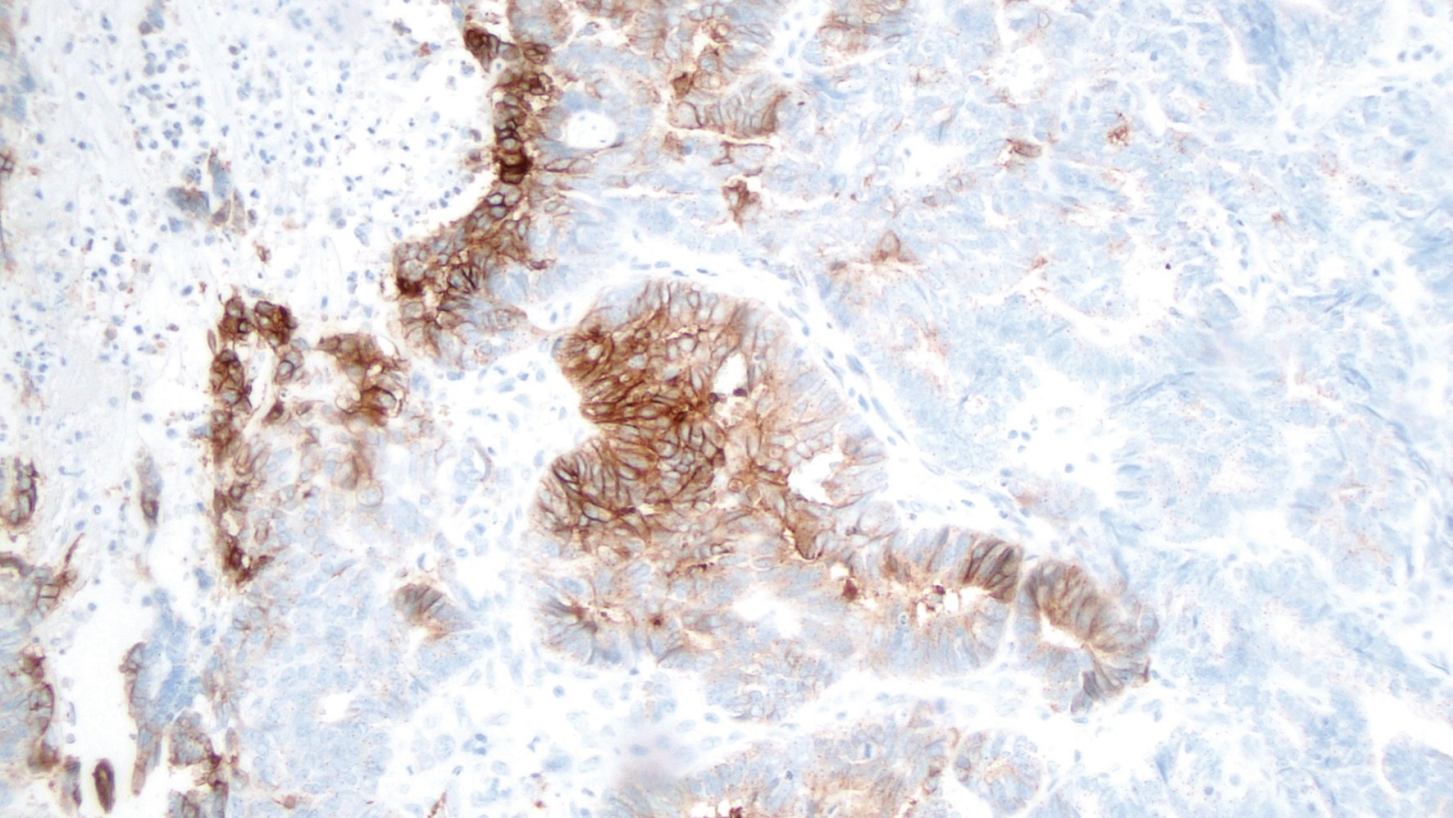
Endometrioid carcinoma with membranous expression of L1CAM.

L1CAM expression was considered positive if the expression was present in 10% or more tumor cells as illustrated in Fig 1.

The tumors were grouped as p53-wild, p53-aberrant, and MMR-deficient (loss of any of the four proteins) according their immunohistochemical profile.

### Statistical analysis

The chi-square test was used to evaluate the association of L1CAM and LVSI with the categorical variables. Age of patients, tumor size, and percentage of myometrial invasion were treated as quantitative variables and compared in the L1CAM, and in the LVSI-positive and negative groups, through the Mann-Whitney U test. Statistical analyses were performed using MedCalc Software, version 17.9.2 (BVBA, Ostend, Belgium). p-values less than 0.05 were considered significant.

## Results

The pathologists were concordant in the interpretation of all cases. No serous or clear cells carcinoma were identified after review. Unequivocal LVSI was identified in 26/41 (63.4%) of the cases submitted to hysterectomy. The involvement was focal in 12 and multifocal in 14 cases. L1CAM was positive in 8/47 (17.0%) cases distributed as follow: 10% of tumor cells (2 cases), 20% (1 case), 30% (1 case), 40% (1 case), 80% (2 cases), and 90% (1 case). All L1CAM-positive cases presented with LVSI (focal in 3 cases and multifocal in 5 cases) and within the high-risk category of ESMO-ESGO-ESTRO. L1CAM expression was associated with FIGO stage >I, high-grade tumors, and immunoprofile p53-aberrant. Seven cases had surgical staging procedure, all of them within the high-risk category of ESMO-ESGO-ESTRO. L1CAM-positive cases presented as larger tumors, with a greater depth of myometrial infiltration, and with a higher frequency of the MELF pattern of infiltration, although these differences did not reach the level of significance. The summary of clinicopathologic characteristic according L1CAM expression is presented in Table 2. The other features associated with LVSI were FIGO stage, tumor size, depth of myometrial infiltration, and tumor grade. Table 3 shows the results of these associations.

**Table 2:** Clinicopathologic characteristics of 47 endometrioid carcinomas of endometrium according L1CAM expression.

	Total	L1CAM-positive	L1CAM-negative	*p*
Age of patients	37–88 (CI95% 58.2–64.2)	52–67 (CI95% 53.62–66.19)	37–88 (CI95% 58.00–68.75)	0.561
FIGO stage				
IA	9 (28.1)	0 (0%)	9 (36%)	0.084
IB	12 (37.5%)	2 (28.6%)	10 (40%)	
II	3 (9.4%)	1 (14.3%)	2 (8%)	
III	8 (24.9%)	4 (57.2%)	4 (16%)	
FIGO				
stage I	21 (65.6%)	2 (28.6%)	19 (76%)	0.021
Stage >I	11 (34.4%)	5 (71.4%)	6 (24%)	
ESMO-ESGO-ESTRO classes**[10]**				
Low	5 (15.6%)	0 (0%)	5 (20%)	0.004
Intermediate	2 (6.2%)	0 (0%)	2 (8%)	
High-intermediate	12 (37.5%)	0 (0%)	12 (48%)	
High	13 (40.6%)	7 (100%)	6 (24%)	
Tumor size	8–70 mm (CI95% 26.09–35.24) Median 30mm	27-60mm (CI95% 27.97–56.12) Median 35.5mm	8-70mm (CI95% 20.70–31.65) Median 29.5mm	0.061
Myoinvasion (%)	0.2%-100% (CI95% 39.66–61.06)	20%-100% (CI95% 21.62–99.19)	0.2%-99% (CI95% 17.58–67.49)	0.098
Myometrial invasion				
<50%	18 (46.2%)	3 (37.5%)	15 (48.4%)	0.087
>50%	21 (53.8%)	5 (62.5%)	16 (51.6%)	
MELF				
Yes	12 (30.8%)	3 (62.5%)	9 (29%)	0.648
No	27 (69.2%)	5 (37.5%)	22 (71%)	
Tumor grade				
G1/2	37 (78.7%)	3 (37.5%)	34 (87.2%)	0.002
G3	10 (21.3%)	5 (62.5%)	5 (12.8%)	
LVSI				
No	15 (36.6%)	0 (0%)	15 (45.5%)	0.018
Yes	26 (63.4%)	8 (100%)	18 (54.5%)	
IHC profile				
MMR	20 (42.6%)	4 (50%)	16 (41%)	0.030
p53-wild	21 (44.7%)	1 (12.5%)	20 (51.3%)	
p53-aberrant	6 (12.8%)	3 (37,5%)	3 (7.7%)	

**Table 3:** Association between clinicopathologic characteristics and lymphovascular space invasion (LVSI).

	Total	LVSI+	LVSI-	*p*
Age of patients	37–88 (CI95% 58.2–64.2)	37–78 (CI95% 56–66)	45–88 (CI95% 58–68.2)	0.828
`				
`	9 (28.1)	3 (13%)	6 (66.7%)	0.023
IB	12 (37.5%)	10 (43.5%)	2 (22.2%)	
II	3 (9.4%)	3 (13%)	0 (0)	
III	8 (24.9%)	7 (30.3%)	1 (11,1%)	
FIGO				
stage I	21 (65.6%)	13 (56.5%)	8 (88.9%)	0.088
stage >I	11 (34.4%)	10 (43.5%)	1 (11.1%)	
ESMO-ESGO-ESTRO classes**[10]**				
Low	5 (15.6%)	0 (0%)	5 (55.6%)	<0.0001
Intermediate	2 (6.2%)	0 (0%)	2 (22.2%)	
High-intermediate	12 (37.5%)	11 (47.8%)	1 (11.1%)	
High	13 (40.6%)	12 (52.2%)	1 (11.1%)	
Tumor size	8–70 mm (CI95% 26.09–35.24)	20-70mm (CI95% 30–39.65)	8-39mm (CI95% 14.06–29.47)	0,0003
Myoinvasion (%)	0.2%-100% (CI95% 39.66–61.06)	5%-100% (CI95% 47.39–82.90)	0.2%-65% (CI95% 8.59–53.62)	0.003
Myometrial invasion				
<50%	18 (46.2%)	8 (30.8%)	10 (76.9%)	0.007
>50%	21 (53.8%)	18 (69.2%)	3 (23.1%)	
MELF				
Yes	12 (30.8%)	10 (38.5%)	2 (15.4%)	0.146
No	27 (69.2%)	16 (61,5%)	11 (84.6%)	
Tumor grade				
G1/2	37 (78.7%)	18 (69,2%)	14 (93.3%)	0.076
G3	10 (21.3%)	8 (30.8%)	1 (6.7%)	
L1CAM				
Negative	33 (80.5%)	18 (69.2%)	15 (100%)	0.018
Positive	8 (19.5%)	8 (30.8%)	0 (0%)	
IHC profile				
MMR	20 (42.6%)	12 (46.2%)	5 (33.3%)	0.247
p53-wild	21 (44.7%)	9 (34.6%)	9 (60%)	
p53-aberrant	6 (12.8%)	5 (19.2%)	1 (6.7%)	

## Discussion

Endometrial carcinomas occur more frequently as the endometrioid histological type, until recently considered the prototype of the estrogen-related carcinomas (type I). This diagnosis is often still restricted to the uterine body and considered as of favorable prognosis. The increasing incidence, together with advances in molecular knowledge, have exposed their heterogeneity and the need for different therapeutic approaches, particularly in terms of endometrioid histology.

Endometrioid carcinomas represent 80% of endometrial carcinomas and patients are often diagnosed at an early stage with the disease localized in the uterus [11]. Most of these cases have a favorable prognosis and could be treated without lymphadenectomy. However, it must be noted that even low-grade endometrial carcinomas can recur [12–14].

The definitions of risk of lymph node involvement, risk of recurrence and of overall survival through morphological analysis are limited. This is partly due to the fact that the criteria are not sufficiently reproductible, but also because there is considerable overlapping between cases of low and high grade. The surgical staging proposed by FIGO is based on the depth of myometrial infiltration, locoregional extension and distance of dissemination [9]. The incorporation of other information such as type and histological grade, patient age, tumor size and LVSI was considered for risk stratification of recurrence in several systems and in clinical trials, such as PORTEC1 (Post-Operative Radiation Therapy in Endometrial Carcinoma) and GOG (Gynecologic Oncology Group) [15]. The American Joint Committee on Cancer in its 8^th^ edition reports the following prognostic factors with level I evidence: FIGO stage, histologic grade and type, depth of myometrial infiltration, LVSI, omentectomy (for high grade tumors), pelvic and para-aortic lymph nodes, peritoneal cytology and morcellation of surgical specimens. Estrogen and progesterone receptors, and expression of suppressor genes and oncogenes, are level II of evidence. However, the definitions of the AJCC prognostic groups, at this date, only include FIGO categories [16].

The consensus conference on endometrial cancer with representatives of the European Society for Medical Oncology (ESMO), the European Society for Radiotherapy & Oncology (ESTRO), and the European Society of Gynaecological Oncology (ESGO) suggested a risk stratification to guide adjuvant therapy based on the International Federation of Gynecology and Obstetrics (FIGO) stage, tumor type, tumor grade, depth of myometrial invasion, and LVSI [10]. The ESMO-ESGO-ESTRO consensus revised in 2014 presents an accurate stratification of risk to guide adjuvant therapy [10]. However, it is primarily based on the FIGO surgical staging, and includes lymph node study.

The possibility of omission of the extensive retroperitoneal lymphadenectomy has been widely discussed [17, 18]. Endometrial carcinomas increase in frequency and affect women with comorbidities associated with obesity, one of the most important risk factors for the disease and for extensive surgical procedures. The inclusion of lymphadenectomy in the treatment requires surgical expertise and reference centers, all of which lead to increased costs. Mariani et al. [19] had already questioned the indication of lymphadenectomy in 2000. On that occasion, they proposed that tumors 20 mm or less were candidates to omission of lymphadenectomy. Besides tumor size, later, the Mayo Clinic nomogram [20] included myometrial invasion, tumor grade, cervical stromal invasion, and LVSI, although it considered an alternative model without tumor size. The tumor size of our study ranged from 8-to 70mm (30.67±13.51) (CI95% 25.00–33.02), with a median size of 30mm. Only 10/36 of our cases (31.2%) measured 20mm or less.

The limitation of these models is the inclusion of variables of the surgical specimen, requiring an intraoperative frozen section examination (FSE) or a two-step surgical procedure. Although FSE is accurate to determine myometrial invasion, tumor size and cervical extension, it is our opinion that it is not feasible for all hospitals and has limitations regarding LVSI [21].

The Cancer Genome Atlas (TCGA) defined four distinct endometrial carcinomas prognostic subgroups, *POLE* ultramutated, microsatellite instability hypermutated (MSI), copy-number-low microsatellite stable (CN-low-MSS), and copy-number-high serous-like (CN-high) [22]. These categories can be determined by relatively simple surrogates: mutations in the exonuclease domain of *POLE*, loss of mismatch repair proteins (MMR) (MLH1, MSH2, MSH6, and PMS2), and p53 expression (aberrant or wild-type) [23]. The integration of molecular classification into pathologic diagnosis was a great advance in clinical practice. This classification is an effective tool to stratify patients according prognosis and, possibly, different therapeutic approaches, particularly when it comes to immunotherapies [24, 25]. The preliminary results with the molecular based classification indicate that the diagnosis through biopsy is highly concordant with the surgical specimens [26]. The prognostic differences in the molecular subgroups determined by the TCGA were observed in conventionally treated patients and, therefore, it is not possible to know if the omission of the lymphadenectomy would have had any influence on the outcome. Moreover, the prognostic curves determined by the MMR e p53-wild groups overlap. Clearly, we need additional markers to personalize treatment.

The inclusion of other biomarkers to improve pre-operatory evaluation, such as L1CAM, seems useful. Others have found significant association between L1CAM and p53-mutant, with evidence of different mechanisms, although both related to risk recurrence [27]. A concern with the use of L1CAM is its heterogeneous distribution, although 10% of positive cells is enough to define its positivity. We support us on data that demonstrated concordance between biopsies and surgical specimens. Tangen et al. [28], performed L1CAM stain in 795 hysterectomy and 1134 curettage specimen, and demonstrate significant correlation. Van Esterik et al. [29] recognize the results of previous studies considering molecular analysis on pre-operative biopsies concordant with the final hysterectomy, and aimed investigate the impact of tumor heterogeneity in the molecular risk assessment in endometrial cancers. They studied 49 cases regarding the expression of *POLE*, *CTNNB1*, p53, MMR and L1CAM. L1CAM concordance was 91.8% (45/49). In this sense, we feel comfortable in studying its expression primarily in surgical specimens. In addition, in 8 of our cases, the study was performed in biopsies because they contained more tumoral volume.

All L1CAM-positive cases of our series presented LVSI. The other variable associated with LVSI were FIGO stage, tumor size, depth of myometrial infiltration, and tumor grade. Except tumor grade, all the variables depend upon the surgical specimens.

It is well stablished [30, 31] that the presence of LVSI is a potent predictor of lymph node metastasis. Even more, it is a powerful prognostic factor, even in the patients with no lymph node metastasis. Tangen et al. [28] demonstrated that expression of L1CAM in curettage was predictive of lymph node metastasis and correlated to L1CAM level in the corresponding hysterectomy.

The small number of cases is a weakness of this study, however, there are some strengths such as, unselected cohort of patients treated in a single cancer reference center and a uniform surgical and pathology team.

Our preliminary results with the expression of L1CAM stimulate its use as a surrogate of LVSI, and so lymph node involvement, with the advantage of possible preoperative evaluation. Although limited by the small number of cases, the results show a rather optimistic trend and may stimulate extension to a larger number of cases.
